# Qualitative and quantitative studies of chemical composition of sandarac resin by GC-MS

**DOI:** 10.1007/s11356-016-7261-5

**Published:** 2016-07-28

**Authors:** I. Kononenko, L. de Viguerie, S. Rochut, Ph. Walter

**Affiliations:** Laboratoire d’Archéologie Moléculaire et Structurale, UMR 8220, CNRS - Université Paris VI, 75005 Paris, France

**Keywords:** Varnish, Sandarac, GC-MS, Diterpenoid

## Abstract

The chemical composition of sandarac resin was investigated qualitatively and quantitatively by gas chromatography-mass spectrometry (GC-MS). Six compounds with labdane and pimarane skeletons were identified in the resin. The obtained mass spectra were interpreted and the mass spectrometric behaviour of these diterpenoids under EI conditions was described. Quantitative analysis by the method of internal standard revealed that identified diterpenoids represent only 10–30% of the analysed sample. The sandarac resin from different suppliers was analysed (from Kremer, Okhra, Color Rare, La Marchande de Couleurs, L'Atelier Montessori, Hevea). The analysis of different lumps of resins showed that the chemical composition differs from one lump to another, varying mainly in the relative distributions of the components.

## Introduction

In his collection of writings ‘A Treatise on Painting’, Leonardo da Vinci proposes a recipe for an excellent varnish based on the cypress resin: ‘...incise a cypress in April or May, mix the exuded liquor with nut oil and get a perfect varnish...’ (Vinci 2003). The cypress resin known as the *sandarac* resin has been widely used throughout history for artistiс purposes as proteсtive varnishes, additives to modulate the rigidity of piсtorial layers and fixatives for artistiс drawing (Romero-Noguera et al. 2014; Scalarone et al. 2005; Merrifield 1849; Watin 1772). There are different types of sandarac varnishes depending on the solvents used (oil-based varnish, spirit of turpentine or alcohol varnishes). Today sandarac is widely used as a resin for the preparation of varnish for musical instruments (Dieterich 1920; Echard et al. 2007).

Leonardo da Vinci pointed out in his text the necessity to use a resin just exuded from the tree at the beginning of spring. So, it is important to distinguish between the fresh and aged resins. In the existing literature concerning this subject, the common misconception takes place. According to the most published materials, the aged resins are those that were aged on purpose by exposing the resin-based varnish to the artificial and non-artificial light (Scalarone et al. 2002, 2003a, b). And the resins that were obtained by means of extraction from a plant and didn’t undergo any specific light treatment are called fresh resins. That can be confusing as the natural resins have tendency to be modified with time when extracted. So, the fresh resins are those that are used at once when exuded from a tree and protected from spontaneous oxidation and polymerisation on air by taking the precautions. The aged resins, in its turn, are those that were exposed to air during some period of time even without being aged on purpose. Obviously, the longer the resin is exposed to the environment the more aged it becomes and consequently harder in its consistency.

**Fig. 1:**
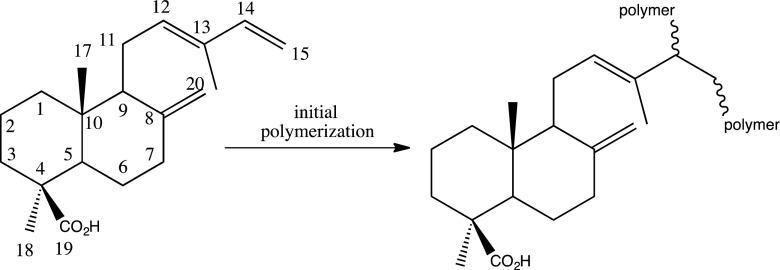
Polymerisation of communic acid

This research aims at analysing by gas chromatography-mass spectrometry (GC-MS), the molecular composition of the sandarac resin, and to give new insights on the markers that can be used for its identification. The sandarac resin sold today by artist’s suppliers is an aged resin, hard, highly oxidised and polymerised and then not suitable for being used as a source for oil varnishes. Although the scientific literature concerning the sandarac, as an art material is limited, it is known that sandarac consists of a polymer fraction and of lower molecular weights compounds, the most characteristic being diterpenoid acids of labdaniс struсture (Romero-Noguera et al. 2014). Such acids with labdane skeletons with its system of conjugated double bonds demonstrate a strong tendency to the polymerisation when executed from trees and readily reticulate as soon as they come into contact with light and air. The exudates of the resin, after having been exposed to the outer atmosphere form a solid protective layer covering the exposed plant tissues (Clifford and Hatcher 1995). Initial polymerisation probably occurs with a radical mechanism starting from conjugated double bonds of labdatriene molecules such as communic acid (Fig. 1).

Moreover, a misconception exists regarding the origin of the sandarac resin. The resin that is commercially available today on the market and called sandarac is the resin that was extracted from cypresses that are planted in the region of North Africa and belong to the family Cupressaceae. Therefore, in this article when using word sandarac, we will be referring to the natural resin obtained from plant belonging to the family of Cupressaceae (*Tetraclinis Articulata*) commercially available but without information on how long the resin was stocked after its actual extraction from a tree.

Several GC-MS as well as pyrolisys-GC-MS studies have been previously made in order to define molecular composition of sandarac resin and presented in the literature. Scalarone’s team applying both techniques accomplished the most complete research on this topic: GC-MS for low molecular weight compound analyses and Py-GC-MS to characterise the composition of polymerised part of the resin (Scalarone et al. 2003a, b). According to them, free diterpenoids identified by GC-MS are sandaracopimaric acid, agathic acid and its monomethyl ester, agathalic acid and acetoxy agatholic acid. *Cis*- and *trans*-communic acids have been detected in traces. Further, to determine the nature of the high molecular weight fraction, the pyrolysis was required. The THM-GCMS (thermally assisted hydrolisys and methylation, coupled with gas chromatography-mass spectrometry) approach was used to identify the cross-linked part. This technique results in a large number of substances that are originated from the pyrolysis process, which is considered to be its main disadvantage. Fragmentation, isomerisation, recombination and other secondary pyrolysis reactions produced a large number of peaks. J.Romero-Noguera et al. have also analysed commercial resin sandarac applying GC-MS analysis and have identified that the composition of sandarac resin is presented by following diterpenoids: manool, sandaracopimaric acid, isopimaric acid and OH-sandaracopimaric acid (Romero-Noguera et al. 2014). As it can be concluded, the results found by two research groups significantly differ, although they were both aimed to analyse the same type of natural resin.

Here, we will first discuss the qualitative analysis of sandarac resin sold by six companies by GC-MS. Gas chromatography-mass spectrometry (GC-MS) is an established technique for the analysis of complex mixtures, holding a prime position in analytical chemistry because of its combination of sensitivity, wide range of applicability and versatility (Lakshmi Hima Bindu et al. 2013; Marinach et al. 2004). Secondly, we will evaluate the quantity of the sample being analysed by GC-MS. The quantitative analysis of sandarac resin is realised by means of an internal standard method that is widely used as an efficient quantitative approach in analytical chemistry **(**Kutnink et al. 1987). An internal standard is a chemical substance that is added in a known amount to the sample in order to calculate the relative quantity of the compounds present in the sample. The internal standard is a compound that is very similar, but not identical to the chemical species of interest in the samples. And, finally, the different sandaracs will be compared in order to conclude how strongly differs the chemical content of the commercial resin from one supplier to another. As mentioned previously and after bibliographic research on the sandarac composition, a contradiction among the presented information on this topic was noticed. While one source reporting on sandarac claimed the presence of agathalic acid as a major component of the resin (Scalarone et al. 2003a, b), the others testified the sandaracopimaric acid being the principal component (Romero-Noguera et al. 2014).

## Materials and methods

The resin studied was the sandarac (*Tetraclinis articulata*) supplied by Kremer, Okhra, Color Rare, La Marchande de Couleurs, L'Atelier Montessori, Hevea. Isopimaric acid (≥98%, GC) was purchased from Sigma-Aldrich.

Gas chromatography experiments were carried out with a GC 5890A gas chromatograph (Hewlett Packard, USA) equipped with capillary column HP-5MS cross-linked 5% Ph Me Silicone (30 m/0.25 mm/0.25 mm) and coupled with a Hewlett Packard GC 5970 mass spectrometer. The GC column temperature conditions were as follows: initial temperature of 40 °C, hold for 2 min, increased at 8 °C min ^−^1 to 150 °C, increased at 3 °C min ^−^1 to 280 °C. Helium gas flow was set at 1 ml min ^−^1. Mass spectra were recorded under electron impact ionisation at 70 eV electron energy, in the range from m/z 40 to 800. Derivatization was done using BSTFA/TMCS (80:20, *v*/*v*) for 1 h at 70 °C. The resulting solution was taken to dryness and dissolved in cyclohexane. Internal standard experiments were accomplished by dissolving 2.5 mg of the derivatized resin in 950 μl of cyclohexane and by subsequent addition of 50 μl of 0.25 mg/ml solution of isopimaric acid in cyclohexane.

## Results and discussion

**Fig. 2:**
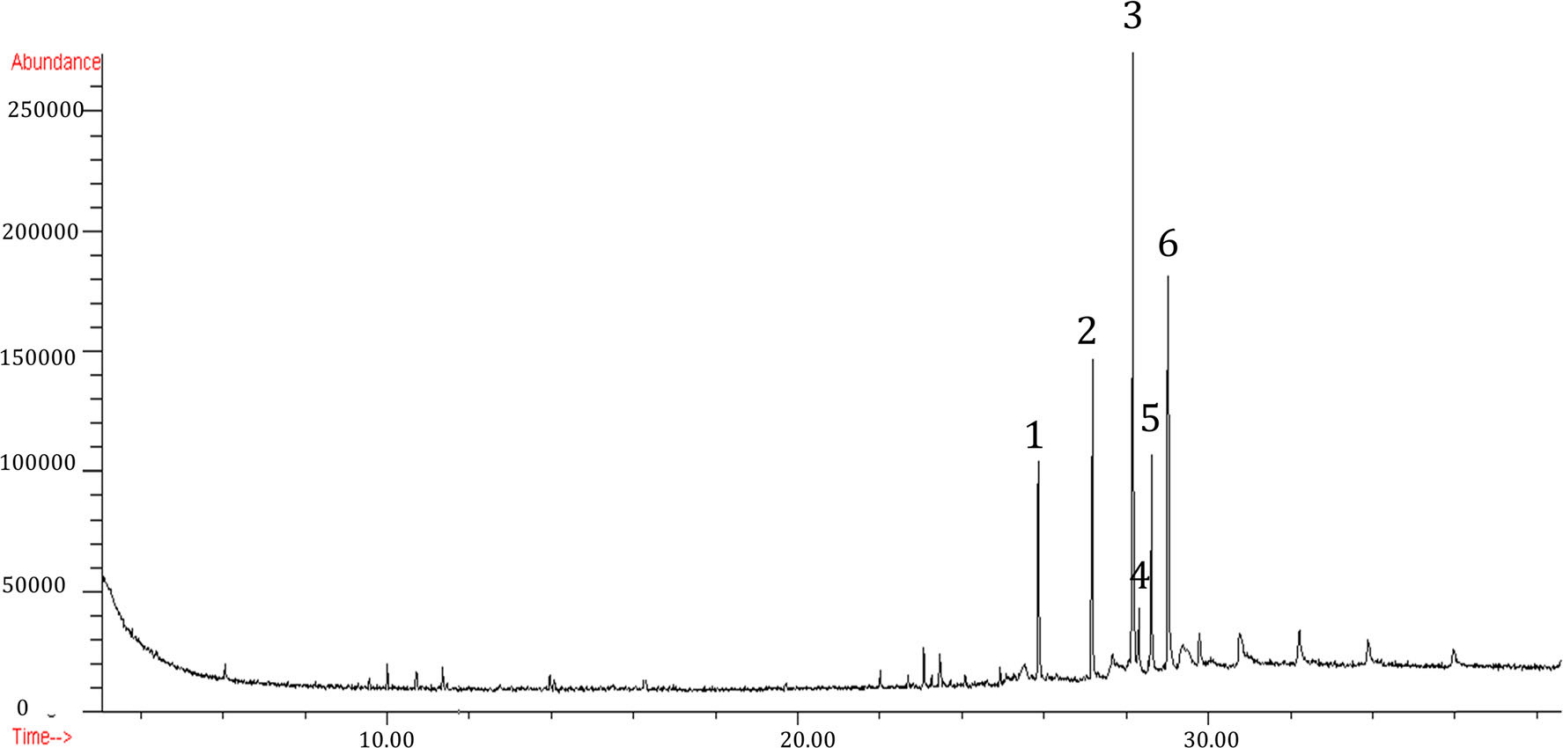
Total ion chromatogram of the sandarac resin after derivatization with BSTFA /TMCS (Kremer)

**Fig. 3:**
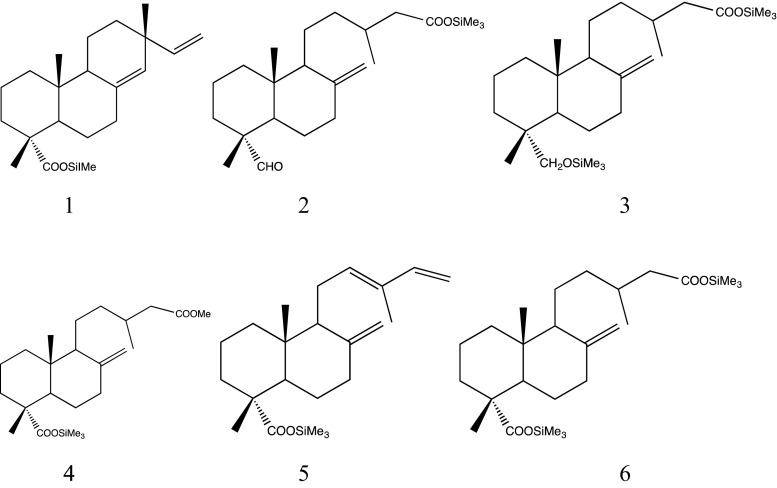
Structures of diterpenoids identified in the sandarac resin by GC-MS

In this study, diterpenoid acids of sandarac were identified by GC-MS, after trimethylsilylation with BSTFA/TMCS. The free diterpenoids identified in the six samples by GC-MS (Fig. 2) and listed in Table 1 are as follows: sandaracopimaric acid (1), dihydroagathalic acid (2), dihydroagatholic acid (3), methyl pinifolic acid (4), communic acid (5) and dihydroagathic acid (6). Interpreting their mass spectra has identified most of the labdanoids present in sandarac (Pastorova et al. 1997; Osete-Cortina and Doménech-Carbo 2005; Berg 2000; Scalarone et al. 2003a, b) (Fig. 3).

**Table 1:** Diterpenoids identified in the sandarac resin by GC-MS and their characteristic fragments

No.	Name of compound	t_R_	M^+.^	[M-CH_3_]^+^	[M-COOSi(CH_3_)_3_]^+^	[M-HCOOSi(CH_3_)_3_-CH_3_]^+^
1	Sandaracopimaric acid, TMS ester	25.864	374	359	257	241
2	Dihydroagathalic acid, TMS ester	27.173	392	377	275	259
3	Dihydroagatholic acid, TMS ester	28.163	466	451	349	333
4	Methyl pinifolic acid, TMS ester	28.303	422	407	305	289
5	Communic acid, TMS ester	28.621	374	359	257	241
6	Dihydroagathic acid, TMS ester	29.027	480	465	363	347
7	Isopimaric acid, TMS ester	26.409	374	359	257	241

**Fig. 4:**
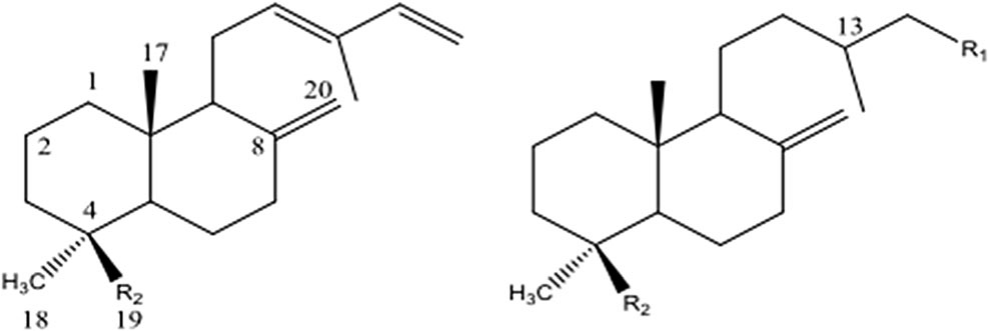
Bicyclical structures of diterpenoids of labdane type

All the diterpenoids of labdane type that were found show a typical bicyclical structure with one or two functional groups situated in the ring A (R_2_) or in the side chain (R_1_) (Fig. 4). Characteristic peaks in the EI-mass spectra result from the molecular ions, from cleavage of the functional groups and from the loss of the side chain. For the sandaracopimaric acid, TMS ester characteristic peaks are the molecular ion at m/z 374 and the fragment ions at m/z 359, obtained by loss of a methyl group [M-15]^+^, at m/z 257 by loss of the trimethylsilyl ester [M-117]^+^ and loss of formic acid trimethylsilyl ester and a methyl group [M-133]^+^.

Although the sandarac resin is still being widely used as a source for varnishes (Azémard et al. 2014), no quantitative analysis was realised. With the study presented hereby, the quantitative assessment of the diterpenoid part has been made for the first time by the method of internal standard. The isopimaric acid was used as an internal standard due to its chemical similarity of the diterpenoids identified in the resin and to its commercial availability. Table 2 lists the diterpenoids identified in the sandarac resin from different suppliers by GC-MS and their relative quantity.

**Table 2:** The quantitative assessment (in %) of each identified diterpenoid by GC-MS

No.	Name of the compound	% of the compound in the sample of resin (2.5 mg)					
		Kremer	Okhra	Color Rare	La Marchande de Couleurs	L’Atelier Montessori	Hevea
1	Sandaracopimaric acid, TMS ester	1.4	2.8	7.6	3.2	7.9	6.5
2	Dihydroagathalic acid, TMS ester	3.1	3.4	2.1	n.d.	5.6	3.6
3	Dihydroagatholic acid, TMS ester	6.7	7.8	4.6	n.d	6.2	3.1
4	Methyl pinifolic acid, TMS ester	7.8	6.2	4.7	0.4	1.1	2.8
5	Communic acid, TMS ester	3.7	3.4	1.8	6.9	2.0	0.1
6	Dihydroagathic acid, TMS ester	7.1	8.3	0.8	0.3	3.7	5.0
	Total percentage	29.8	31.9	21.6	10.8	26.5	21.1

*n.d.* not detected

Although relative amount of each particular diterpenoid varies in a wide range depending on the supplier, it comes evident from the table that almost all sandaracs except that from *La Marchande de Couleurs* consist of the same diterpenoids. The sandarac purchased from *La Marchande de Couleurs* stands out exceptionally in comparison with other suppliers. Dihydroagathic and dihydroagatholic acids are not identified in their sandarac at all. Methyl pinifolic and dihydroagathic acids in its turn are found only in traces. On the other hand, the sandarac from *La Marchande de Couleurs* has the highest concentration of communic acid. Indeed, in all other five sandaracs the percentage of communic acid doesn’t exceed 4%. Meanwhile, this value in case of *La Marchande de Couleurs* is almost twice bigger. For the other resins, the qualitative composition is the same: all six diterpenoids are found in the rest five samples.

**Fig. 5:**
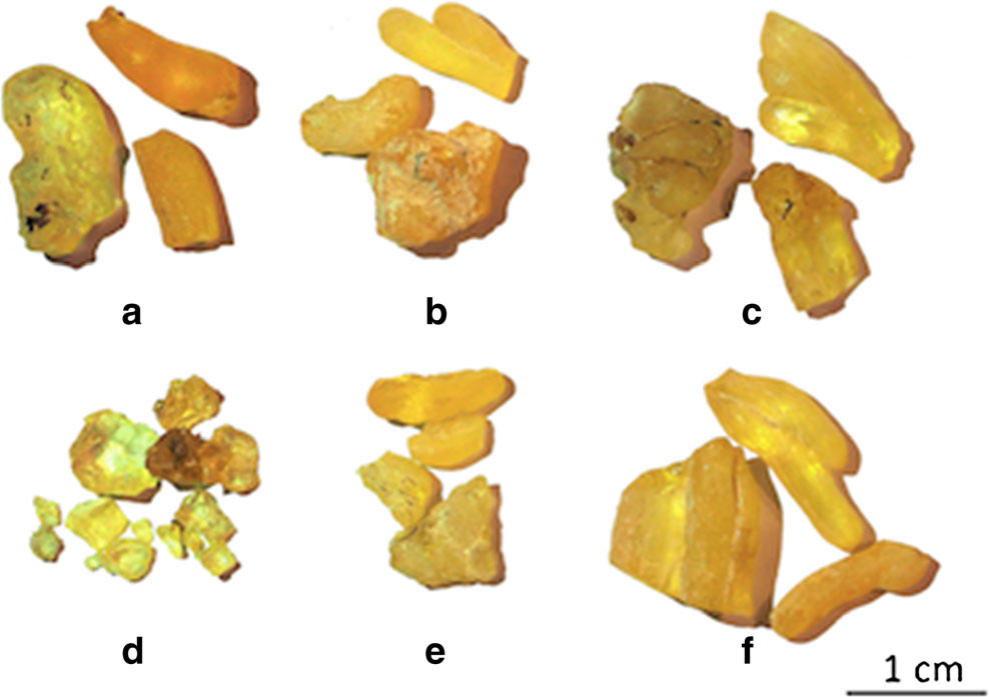
Lumps of the resins from six different suppliers (Kremer (**a**), Okhra (**b**), Color Rare (**c**), La Marchande de Couleurs (**d**), L'Atelier Montessori (**e**), Hevea (**f**))

The Fig. 5 presents the lumps of the resins from six different suppliers to show how the resin solidifies when exposed to the air and comes to commerce in the form of small solid chips, translucent, and having a delicate yellow tinge. The lumps of resin from five suppliers, having similar chemical composition, are visually close as well, while the resin from La Marchande de Couleurs, standing out with its particular composition, shows different shade and shape properties.

It is remarkable that sandarac resins delivered by *Kremer* and *Okhra* have very close quantitative composition. They are both characterised by weak concentration of sandaracopimaric and communic acid, and at the same time by relatively high percentage of agathic acid’s derivatives. The overall percentage of diterpenoids present in the resins from these two suppliers reaches 30%, which is the highest concentration of the diterpenoid acids among the other resins. The total amount of pimaranes and labdanes in the case of *Color Rare*, *L’Atelier Montessori* and *Hevea* ranges in the average of 20–25%. And eventually, *La Marchande de Couleurs* stands here alone as well, showing the presence of only 10% of diterpenoids.

Previous research on the chemical composition of the sandarac resin, as outlined earlier in the introduction, showed different results presenting diterpenoids that were not identified here. The only compound that was found in common was sandaracopimaric acid. Compounds such as manool, isopimaric acid or OH-sandaracopimaric acid were not found in the six samples analysed here, whereas they have been found by name (Romero-Noguera et al. 2014). The reason for such radical difference in results might be hidden in different methods of resin’s derivation. Indeed, GC-MS analysis on the sandarac resin was accomplished after methylation of samples with trimethylammonium hydroxide that produced diterpenoid’s methyl esters. In the current study, the trimethylsilylation was chosen as the method of sandarac derivation. Anyway, it remains an open question if two different ways of the resin derivation could lead to such a great difference in results for the same type of resin.

Following the hypothesis widely supported in the literature that the communic acid is the first diterpenoid to be responsible for the polymerisation of the sandarac resin, the sandarac from *La Marchande de Couleurs* is the freshest resin among the others. That means that the sandarac from this supplier still contains communic acid in an excessive quantity relatively to other analysed resins. Therefore, the sandarac resin purchased from *Hevea* might be the oldest one having the lowest percentage of communic acid that had probably completely polymerised.

Lacking the information on the actual origin of the resin purchased, on the method and conditions of its treatment, it is difficult to judge if the difference in chemical composition of the sandarac (mainly quantitative) is due to the difference of trees’ species and if an important factor can be the time during which the resin was stored. Actually, even the fact that the resin is originated from the same tree species is not yet the guarantee of the identical chemical composition of its resin.

The results presented in the Table 2 demonstrate that the total amount of the diterpenoids that we can analyse by this method is between 10 and 30 %, depending on the sample. The rest of the sample is probably a polymerised fraction of the resin, proving once again the fact that the sandarac is a highly reticulated resin.

## Conclusions

Qualitative and quantitative analyses by gas chromatography-mass spectrometry (GC-MS) were accomplished for identification of the chemical composition of the sandarac resin. GC-MS has been applied to a sample of a natural resin sandarac as a well-suited technique for the recognition of small molecules. The method of internal standard was applied in order to quantify and assess the sample’s diterpenoid content. As a result of quantification, the non-polymerised fraction of the sandarac resin is less than 1/3 of the sample in total.

Six compounds with labdane and pimarane skeletons were identified in the resin. It has been demonstrated as well that the quantitative chemical composition of sandarac resin differs from one supplier to another, while the qualitative composition is almost the same. Sandaracopimaric and communic acids are present in all resins. The rest of the diterpenoids is found in the different sandarac analysed with various proportions. Dihydroagathic acid was not identified in the sandarac from *La Marchande de Couleurs*, but communic acid is present in a larger quantity compared to other resins. The sandarac from *L'Atelier Montessori* demonstrated only traces of methyl pinifolic and dihydroagathic acids. We show here a wide range of composition for resins from different suppliers, which can explain the difference existing in the literature concerning sandarac. The contradiction that appeared in the two previous studies (Romero-Noguera et al. 2014, Scalarone et al. 2003a, b) can be explained by this variability. Another explanation would rely on the potential influence of the resin derivation method. In order to deeper the existing knowledge, it would also be interesting to investigate the true impact of the derivation methods.

Systematic analyses on the natural products that are sold to artists on the market can provide them with a better insight on the materials they are using. Of course, the composition of natural products on the molecular level may differ notably depending on many factors such as geographic and climatic; differences that would impact drastically the artist’s work. That is why artists prefer to stay with one particular art supplier.
